# Comparison of metal burden in different muscle tissues of Great cormorant (*Phalacrocorax carbo*)

**DOI:** 10.1007/s11356-021-15600-z

**Published:** 2021-08-09

**Authors:** József Lehel, Adrienn Grúz, András Bartha, Imre Pintér, Zoltán Lénárt, László Major, László Menyhárt, Rita Szabó, Péter Budai

**Affiliations:** 1 Department of Food Hygiene, University of Veterinary Medicine, István u. 2, Budapest, H-1400 Hungary; 2 Institute of Plant Protection, Georgikon Campus, Hungarian University of Agriculture and Life Sciences, Deák F. u. 16, Keszthely, H-8360 Hungary; 3 Herd Health and Mobile Clinic, Department of Animal Hygiene, University of Veterinary Medicine, István u. 2, Budapest, H-1400 Hungary; 4 Division of Plant Protection and Soil Conservation, Department of Agriculture, Government Office of Jász-Nagykun-Szolnok County, Vízpart blvd. 28, Szolnok, H-5000 Hungary; 5 Institute of Technology, Georgikon Campus, Hungarian University of Agriculture and Life Sciences, Deák F. u. 16, Keszthely, H-8360 Hungary

**Keywords:** Great cormorant, Pectoral muscle, Thigh muscle, Metals, Heavy metals, Wetland, Metal pollution

## Abstract

Concentrations of 12 metals (As, Ba, Cd, Co, Cr, Cu, Hg, Mn, Mo, Ni, Pb and Zn) were examined in the pectoral and thigh muscle of great cormorants (*Phalacrocorax carbo*). The samples were collected from Central Tisza-Jászság Nature Conservation Area in Hungary. The tissue samples were analysed by inductively coupled plasma optical emission spectroscopy (ICP-OES). The aim of the study was to examine the impacts of heavy metal pollution on the water birds, determine the concentrations of the abovementioned metals in the different muscle tissues of these wetland birds, and provide the basic materials for monitoring the environmental pollution. Among the investigated elements/metals, the detected concentrations of As, Ba, Cd, Co, Cr, Mo and Ni were below the detection limit. Higher concentration of Cu, Hg, Mn and Pb was measured in the pectoral muscle compared to the thigh muscle, but only in the case of Cu and Mn were found significant differences between the tissues. In the case of the Zn concentration, the higher value was detected in the thigh muscle. There were no statistical differences between males and females in either metal concentrations.

## Introduction

Due to the rapid development of toxicology, more and more researches and studies have been published that have reported the pronounced toxicity of various compounds of metals. Based on the results, the use of many metal compounds as pesticides has been banned by the licensing authorities. However, due to anthropogenic activities (widespread use of metals and their continuous industrial emissions, industrial and agricultural wastes, mining activities) and urban pollution (e.g. incineration of municipal solid waste, road dust, heating), the entire biosphere has become contaminated in different levels (EFSA 2009; Alipour et al. 2013). At the same time, they can enter the circulation from natural sources by erosion, sedimentation, and decomposition as well. Thus, currently, particularly due to the polluting effect of metals and their enrichment in the food chain because of their cumulative properties, they are a very important aspect in the protection of the environment. To be able to evaluate the level of hazard and the degree of exposure to any metal contamination, it is necessary to carry out biological monitoring using biological indicator species, e.g. wild birds (Furness and Greenwood 1993). The metal burden in the body of the birds has influence on the breeding, the growth, the moulting and migration of the birds (Hutton 1981; Honda et al. 1985).

Different bird species, e.g. mallards (*Anas* spp.), cormorants (*Phalacrocorax* spp.), gulls (*Larus* spp.), white storks (C*iconia ciconia*), common buzzard (*Buteo buteo*), feral pigeon (*Columba livia domestica*) and eagle owl (*Bubo bubo*), which are located on higher trophic levels in the ecosystem, are widely used as bioindicators for monitoring metal contamination in the environment (Blus et al. 1993; Bearhop et al. 2000; Ortego et al. 2006; Guitart et al. 2010).

Metals can usually enter organisms through the digestive tract, respiratory tract and integument and absorbed to varying degrees depending on their physicochemical properties, size, lipid solubility and ionization. They must cross through different biological membranes as they move within the body. For the transportation, membrane transport proteins are needed. These proteins behave as carriers or channel molecules in the cell membrane. For most metals, these proteins have been identified already. These can be quite selective, due to selective binding sites that recognize specific chemicals, so transporting only one metal species but not another. The absorbed metals enter the bloodstream and are transported to organs and tissues. Metals absorbed from the gastrointestinal tract first enter the liver by the portal circulation and enter the systemic circulation only after passing through the liver. In the blood, they mostly attached to red blood cells or various plasma components; e.g. Pb, Mn and organic mercury compounds are transported by red blood cells, while inorganic Hg compounds, Cu, Cd and Zn, are attached to the albumin. During distribution, compounds with high affinity for a tissue may bind to them, which can lead to accumulation. In the case of various metals, e.g. Hg, the accumulation may occur not only in the body of the organism, but in the food chain as well (biomagnification). Metals are especially excreted via urine and faeces (through biliary clearance), but other routes may be probable (Lehel and Laczay 2011).

The metals in the process of absorption, distribution and redistribution can cause morphological or neurological changes (Kim and Oh 2013), and the difference in the level of distribution and accumulation in the tissues can be analysed using the kidneys, liver, muscle tissues, feathers, bones, eggs and excrement of the birds (Burger and Gochfeld 2000a; Markowski et al. 2013).

The strongest members of the bird muscles are the wing-moving pectoral muscles, which make up more than half of the total body muscle mass, but in smaller, well-flying birds (e.g. swallows) this proportion can be as high as 80% (Bakonyi et al. 2003). The pectoral muscles of chickens are frequently referred to as “white meat”, because chicken do not fly, so the activity of these muscles is minimal, compared to the sustainedly used thigh muscles, which are referred to as “dark meat”, because it contains a large number of myoglobin-containing cells, which derives its characteristic darker colour to these muscles. This protein functions as an oxygen-storage unit in the cells, providing oxygen to the working muscles. Since cormorants and other birds are capable of flight, their pectoral muscles are dark as well (Jacob and Pescatore 2013; Gill et al. 2020). However, within a muscle, there are more types of fibres. Each muscle also has red and white muscle fibres, the proportions of which also determine the nature of each muscle. In addition, there are so-called also intermediate muscle fibres, which indicate the transition between red and white ones (Fehér 2004).

In our study, we aimed to investigate the heavy metal pollution (and essential metal content) of the pectoral and thigh muscle of great cormorants (*Phalacrocorax carbo*) at area of the Central Tisza-Jászság Nature Conservation Area of the Hortobágy National Park Directorate, and thus the possible metal pollution in the area, and to obtain information, similarity or difference on metal accumulation in the two types of muscle tissues.

## Materials and methods

### Material

In late January of 2020, in the Central Tisza (on the river section below the dam near Kisköre), under the supervision of the Nature Conservation Service, twenty cormorants were shot as a population management activity to reduce the overall cormorant numbers in the region. These birds were collected after being shot with official permission (nature conservation permit of the county government office, No JN-07/61/00253-4/2020), based on Decree No. 13/2001 of the Minister of Environment (2001), because this species induces economical losses in the fish industry and is therefore non-protected. At the necropsy, the experts of the Nature Conservation Service determined the age and sex of the birds, and analysed the stomach contents.

Samplings from pectoral and thigh muscles of 20 cormorants including of both sexes with the same ratio (10 males, 10 females) were taken during the necropsy as well. Twenty grams of both muscle tissue samples was placed into an individually labelled plastic bag. All samples were placed in a cooler bag and transported to the laboratory where they were stored at −20°C until assayed.

### Method

#### Laboratory processing and measurements

The heavy metal concentration of the samples was determined in the analytical laboratory of the Department of Animal Hygiene, University of Veterinary Medicine, using a Perkin Elmer Optima 3300 DV inductively coupled plasma optical emission spectrometry (ICP-OES) as described by Grúz et al. (2017). During the analysis, the following measurement parameters were applied: RF generator, 40 MHz; RF power, 1500W; nebulizer type, concentric (Meinhard Type A); nebulizer gas flow rate, 0.9 dm^3^/min; cooling water flow rate, 1 dm^3^/min; sheath gas flow rate, 0.9 dm^3^/min; sample feeding flow rate, 0.9 cm^3^/min; and observation height, 15 mm.

Nitric acid (HNO_3_) and hydrogen peroxide (H_2_O_2_) and a microwave digester were used to extract the samples.

#### Analytical standards used in sample processing

Calibration was performed with ICP multi- and mono-element standards (Perkin Elmer Inc. USA; VWR International Ltd., England). The measurements were performed with argon gas of 4.6 purity (Messer Hungarogáz Kft).

#### Sample preparation

For sample digestion, 0.5 g from each sample was weighted into a CEM MARS6 MARSXPreSS Teflon vessel. Then they were decomposed by 5 ml nitric acid (69m/m%) and 5 ml hydrogen peroxide (30m/m%) in a microwave digestion system (Ramp: 35 min; temperature: 200°C; hold: 50 min; E: 1700 W). The sample was filled up to 25 ml and then diluted twice.

The following metals were determined from the samples: arsenic (As), barium (Ba), mercury (Hg), cadmium (Cd), cobalt (Co), chromium (Cr), manganese (Mn), molybdenum (Mo), nickel (Ni), copper (Cu), lead (Pb), zinc (Zn). During the measurement, the limit of detection (LOD) of each metal was as follows: 0.05 μg/g for Cd, Co, Cr, Cu, Mn, Zn; 0.2 μg/g for Ni, Pb; 0.5 μg/g for As, Ba, Hg, Mo.

### Statistical methods

Statistical analyses were performed using SPSS (version 25.0).

Given that in many cases we have censored data (censored data: instead of a specific value, it is mentioned that “below the limit of detection”), sex comparisons were performed using the Mann-Whitney test. In the comparisons, due to the large number, a Benjamini-Hochberg correction was applied to the *p* values. This regulates that no more than 5% of the pairs found to be significant are misclassified. After the correction, there was no significant difference between the sexes in either case.

As there was no significant difference between the sexes in either case, the 10 female and 10 male birds were treated together, as a sample of 20 elements. Due to the censored data, the comparison of tissue samples was performed with a nonparametric Friedman test. This compares the data of an exact tissue sample with all the other tissue samples of the same bird. Pairwise comparisons were then performed using the Wilcoxon test using Bonferroni correction for each body part separately.

## Results

### Stomach content

In the stomach content, the following fish species were found in the highest quantity: two carnivorous species, Zander (*Sander lucioperca*) and Wels catfish (*Silurus glanis*), and two omnivorous species, Danube streber (*Zingel streber*), and Black bullhead (*Ameiurus melas*).

### Evaluation of metals

The average concentrations of metals in birds of both sexes are presented in Table 1.

**Table 1:** Metal concentration measured in breast (pectoral) and thigh muscle (μg/g)

**Sexes**		**As**	**Ba**	**Cd**	**Co**	**Cr**	**Cu**	**Hg**	**Mn**	**Mo**	**Ni**	**Pb**	**Zn**
**Pectoral muscle**													
Females (*N*=10)	Average±SD	0.6±0.1	<0.5	0.1	<0.05		7.2±1.4	0.9±0.3	0.7±0.2	<0.5	<0.2	0.5±0.2 (1.1± 1.9)*	14.8±2.5
	Range	<0.5–0.7	–	<0.05–0.1	–	–	5.5–9.8	<0.5–1.4	0.4–1.0	–	–	0.2–6.3	11.5–20.2
Males (*N*=10)	Average±SD	<0.5	<0.5	0.1	<0.05	<0.05	6.5±1.0	1.1±0.5	0.7±0.2	<0.5	0.3	0.4±0.1	16.1±6.4
	Range	–	–	<0.05–0.1	–	–	4.6–8.3	<0.5–1.2	0.5–1.2	–	<0.2–0.3	0.2–0.6	10.6–33.0
*P* value		0.2	–	1.0	0.7	–	0.7	1.0	1.0	–	0.7	0.7	1.0
**Thigh muscle**													
Females (*N*=10)	Average±SD	0.9	<0.5	0.1±0.0	<0.05	0.1	2.8±0.7	0.8±0.3	0.4±0.1	<0.5	0.3±0.1	0.3±0.2 (6.0±12.6)*	16.6±5.7
	Range	<0.5–0.9	–	<0.05–0.1	–	<0.05–0.1	1.6–4.2	<0.5–1.4	0.3–0.7	–	<0.2–0.4	<0.2–28.6	10.5–28.8
Males (*N*=10)	Average±SD	<0.5	<0.5	<0.05	<0.05	<0.05	3.1±1.1	0.9±1.1	0.5±0.1	<0.5	0.2±0.0	0.5±0.3	17.9±4.3
	Range	–	–	–	–	–	1.7–5.2	<0.5–1.8	0.3–0.6	–	<0.2–0.3	<0.2–1.3	10.1–24.9
*P* value		0.7	–	0.2	–	0.7	0.8	0.8	0.6	–	0.8	0.5	0.7

*Outliers omitted

The measured average concentrations of As, Ba, Cd, Co, Cr, Mo and Ni were under the detection limit. In the case of Cu, Hg, Mn, Pb and Zn, the majority of the individual findings (Cu 100%, Hg 65–80%, Mn 100%, Pb 65–100%, Zn 100%) and the mean concentrations were above the LOD, and were higher in the pectoral muscle, than in the thigh muscle (except the Zn concentration) (Table 2).

**Table 2:** Concentration of investigated metals in the muscle (average±SD) (μg/g)

Sample	Concentration of metal (average±SD) (μg/g)				
	Cu	Hg	Mn	Pb	Zn
Pectoral muscle	6.9±1.2	1±0.4	0.7±0.2	0.4±0.1	15.5±4.6
Thigh muscle	3±0.9	0.9±0.3	0.4±0.1	0.4±0.1	17.3±4.8

### Comparison of sexes

No gender differences were found for pectoral and thigh muscle. During the examination of the pectoral and thigh muscles, outstanding lead concentration was detected in 1–1 cases. Excluding them from the evaluation, there was no gender difference.

### Comparison of the two muscle tissue samples

Significant difference was obtained for each element, suggesting that each metal accumulates in different concentrations in different type of muscle tissues. Among the investigated metals, the detected concentrations of Cu and Mn were significantly higher in the pectoral muscle than in the thigh muscle (Table 3).

**Table 3:** Comparison of muscle samples by Wilcoxon test using Bonferroni correction

Comparison of muscle sample	Adjusted *P* value	
Thigh muscle vs pectoral muscle	Cu	Mn
	<0.001	<0.001

## Discussion

Among the investigated elements/metals, the detected concentrations of As, Ba, Cd, Co, Cr, Mo and Ni were below the LOD. The measured values of Cu, Hg, Mn, Pb and Zn were above the limit of detections; thus, they are discussed.

### Copper (Cu)

Cu is essential in low concentration for the normal growth, cell metabolism, haemoglobin formation, antioxidant defence system and structure and the function of many proteins (Harms and Buresh 1987; Pesti and Bakalli 1996; Underwood and Suttle 1999; Malik and Zeb 2009); however, chronic uptake has many toxic effects on birds including the reduced growth rates and egg production, and developmental abnormalities (Jackson and Stevenson 1981; Chiou et al. 1999). In most animals, the muscle tissues contain about 4 mg Cu/kg dry matter (DM), and will not increase, even on high-copper diet (NRC 2005). In our study, the average concentration of Cu in pectoral muscle was 6.9±1.2 μg/g dry wet (dw) and in thigh muscle 3±0.9 μg/g dw. These concentrations are lower compared to the concentrations found in the literature (Table 4). According to the statistical comparison, the concentration of Cu measured in pectoral muscle is significantly higher than that in the thigh muscle (*P*<0.001). Generally, there is lack of information in the literature about the comparison of Cu in both muscle tissues. However, Carpene et al. (1995) noted that the concentration of Cu was twice as high in the pectoral muscle of black-headed gulls (*Chroicocephalus ridibundus*) than in its thigh muscle. Since the structure of these muscle types are similar, the size of them can be an explanation of the higher metal content, but more specific further examinations are needed to be carried out in this field.

**Table 4:** A comparison of different metal concentrations in muscle tissues of similar wetland species found in the literature with the measured metal concentrations in this study

**Bird species**		**Heavy metal**	**Pectoral muscle** **(breast) (μg/g dw)**		**Thigh muscle** **(leg skeletal) (μg/g dw)**	**Reference**
**Common name**	**Latin name**					
Great cormorant	*Phalacrocorax carbo*	Hg	2.3±2		–	Aazami et al. 2011
Great cormorant	*Phalacrocorax carbo*	Cu	42.6		–	Aazami and KianiMeh 2017
		Hg	2.3		–	
		Zn	71.9		–	
Great cormorant	*Phalacrocorax carbo*	Hg	1.3±0.4		–	Saeki et al. 2000
Great cormorant	*Phalacrocorax carbo*	Hg	1.1±0.3 (ww)		–	Nam et al. 2005
Great cormorant	*Phalacrocorax carbo*	Hg	1±0.3		–	Houserova et al. 2005
Grey heron	*Ardea cinerea*	Cu	49.3±12.3		–	Horai et al. 2007
		Hg	0.4±0.4		–	
		Zn	74.0±13.1		–	
Great white egret	*Ardea alba*	Cu	17.4±2.6		–	
		Hg	0.2±0.3		–	
		Zn	62.1±7.3		–	
Intermediate egret	*Ardea intermedia*	Cu	14.4±2.6		–	
		Hg	0.03±0.03		–	
		Zn	64.6±10.3		–	
Great cormorant	*Phalacrocorax carbo*	Cu	21.7±1.7		–	
		Hg	0.01±0.001		–	
		Zn	56.0±6.90		–	
Cattle egret	*Bubulcus ibis*		Rural area	–		Yasmeen et al. 2019
		Cu	12.6±0.0	–	–	
		Hg	0.1±0.0	–	–	
		Mn	3.4±0.0	–	–	
		Pb	0.1±0.0	–	–	
		Zn	30.9±0.0	–	–	
Neotropic cormorant	*Phalacrocorax brasilianus*	Pb	0.4–1.2 (ww)		–	Cid et al. 2009
Great grebe	*Podiceps major*	Pb	0.3–3.2 (ww)		–	
Black-headed gulls	*Chroicocephalus ridibundus*	Cu	14.3 ± 1.5		7.1 ± 1.7	Carpene et al. 1995
		Zn	45.0± 7.5		99± 22	
Herons and egrets (pooled sample)	*Ardea cinerea* *Egretta alba* *Egretta intermedia* *Egretta garzetta* *Bubulcus ibis* *Nycticorax nycticorax*	Mn	12.5 ± 15.9		–	Kim et al. 2009
		Pb	2.3 ± 2.9		–	
		Zn	52.5 ± 56.5		–	
Great cormorant	*Phalacrocorax carbo*	Cu	6.9±1.2		3±0.9	This study
		Hg	1±0.4		0.9±0.3	
		Mn	0.7±0.2		0.4±0.1	
		Pb	0.4±0.1		0.4±0.1	
		Zn	15.5±4.6		17.3±4.8	

*ww* wet weight

### Mercury (Hg)

Among heavy metals, mercury is one of the most toxic and persistent elements entering the aquatic ecosystem. Wetlands are important habitats for mating and foraging of many wildlife species but are threatened by Hg pollution. In aquatic systems during various biological processes, inorganic mercury converted to methylmercury by anaerobic bacteria in the sediment. Methylmercury is highly toxic, persistent and bioaccumulative; it can be biomagnified in the aquatic food chain (Bloom 1992; Nguyen et al. 2005), so the species on any trophic level can be chronically exposed to Hg. Fish-eating birds, like cormorants, consume fish from the top of the aquatic food chain and receive the methylmercury that has accumulated through the biomagnification process and in their habitat as well (Lavoie et al. 2013). As known, in fish, the methylmercury is accumulated mainly in the muscle tissues, while inorganic mercury is in the gastrointestinal epithelium. Accumulation of mercury in tissues is a slow process, so usually older animals have higher levels than younger animals. Once it has accumulated in tissues, its reduction is very slow, even consuming clean food and water. It has been examined that regardless of the form of Hg, the tolerable dietary levels for animals appear in levels in muscle tissues that may occur toxicosis in humans (NRC 2005). Higher methylmercury uptake can result in adverse behavioural, developmental, neurological, hormonal and reproductive effects (Scheuhammer et al. 2007). Many studies have been published focusing on the Hg pollution accumulated in the aquatic ecosystem, and in the body of wetland bird species. The measured Hg concentrations in the pectoral and thigh muscle of cormorants found in different studies are similar to the concentration found in our experiment (Table 4).

### Manganese (Mn)

Manganese is an essential element and one of the least toxic of the essential elements (NRC 1995). The accumulation of excess manganese in the body is prevented by the gut (ATSDR 2000). Absorbed manganese excreted via the bile very rapidly, even 1 h after ingestion (Malecki et al. 1996). Different dietary factors affect the absorption of manganese, e.g. the presence of calcium and phosphorus. High levels of dietary calcium and phosphorus increase the symptoms of manganese deficiency in chickens (Wilgus Jr and Patton 1939). Organisms on lower levels of the food chain (e.g. plankton, aquatic plants and some fish species) can accumulate Mn in higher concentration, but the potential of biomagnification from lower trophic levels to higher ones was not observed (ATSDR 2012). Mn concentration in the muscle tissue of wetland birds is less documented, but the data found in few studies were higher than the measured concentration in our study (Table 4). Significant differences were found between the two muscle tissue types, and measured higher value in the pectoral muscle, than in the thigh muscle (*P*<0.001). Similar comparison was not found in the literature, likewise in the case of Cu.

### Lead (Pb)

The main source of lead for waterfowl and other wetland species are the various lead shots, and lead weights used in sport fishing (Burger and Gochfeld 2000b; De Francisco et al. 2003). Many bird species take these as crushed stone to aid digestion. These lead-containing formulas remain in the gizzard and dissolve at acidic pH, which results in a continuous lead load. But if the bullet gets into the muscle, mostly it does not cause poisoning because in an alkaline medium it does not dissolve but encapsulates. Unlike mercury, biomagnification of lead is not typical in the environment. In aquatic ecosystem, the highest lead concentrations can be measured usually in benthic organisms and algae, and in species on lower trophic level, e.g. carnivorous fish (NRC 2005). Its main sources of uptake are contaminated soil and lead-contaminated drinking water (Waldner et al. 2002). A higher dose of lead ingested with food is better tolerated by the body than if it is taken up with contaminated water (Galey et al. 1990). The efficiency of lead absorption depends on the chemical form of the lead, the consumed nutrients and the physiological condition of the animals. For example, calcium and phosphate are effective in reducing lead absorption (Varnai et al. 2001). Symptoms of acute poisoning can develop in animals from the consumption of contaminated food, the remains of an animal killed with lead-containing bullets (Galey et al. 1990). Water hardness, pH, salinity and organic matter influence the toxicity of lead-contaminated water. The body burden of lead also effected by the age of the organisms; young animals are more sensitive, but muscle tissues do not accumulate larger quantity of lead (except at very high doses) and after several weeks when it reached its maximum in an organ, it began to decline (NRC 2005). There are only few studies in the literature that measured Pb concentration in pectoral and thigh muscle of piscivorous bird species, but the found ones show similar results to ours (Table 4).

### Zinc (Zn)

Zinc is an essential element, and it is required for DNA replication, transcription and a cofactor for gene regulatory proteins (Malik and Zeb 2009), but it may be toxic if accumulated in tissues of birds in high concentrations. Even if it is relatively nontoxic to birds and mammals and it is more tolerable in higher concentration than in other metals, toxicosis occurs at the dietary concentration in excess of 1000 μg/g. The absorption of Zn is determined by the amount of zinc in the animal’s body, the total amount of the zinc in the diet and its intestinal solubility, which is influenced by the chemical form and the presence of the required inhibitors (Baker and Ammerman 1995; Lonnerdal 2000). In general, large amounts of iron, calcium and phytate present in the body reduce the absorption of zinc, while certain amino acids (e.g. histidine, cysteine) increase it (NRC 2005). The skeletal muscle and bone accumulate about the 85% of the total body zinc (O’Halloran 1993). The measured concertation in our study was lower than the data found in the literature, for both types of the tissues (Table 4). However, Carpene et al. (1995) measured twice as high of Zn concentration in the thigh muscle of black-headed gulls (*Chroicocephalus ridibundus*) than in its pectoral muscle. Even if the concentration in thigh muscle was higher than that in the pectoral, opposite like in the case of the other metals, these results were not significantly different.

The highest metal concentrations of the wild birds are certainly due to the environmental pollution, and thus due to the metal content of their nutrition. Even if the metal concentration is low in the different surface waters, due to their persistence and bio-accumulative property, they can pollute fishes and other members of the aquatic life (Eisler 1988). Many factors, such as the solubility, bioavailability, feeding behaviour, species, age, size, reproductive state, fish health and habitat of the species, affects the concentration of metals accumulate in the different parts of fish’s body (Cross et al. 1973; Lawrence and Mason 2001; Perugini et al. 2014; Anandkumar et al. 2017). According to several studies, in omnivorous species, higher trace element concentrations can be measured (Cheung et al. 2008; Cheng et al. 2016). Jia et al. (2017) found, comparing carnivorous and omnivorous species, that in the case of Cu, Mn and Zn, the concentration was higher in omnivore fishes, while the Cd and Pb were in higher concentration in carnivorous species. Hosseini et al. (2015) had similar observation, that carnivorous fish accumulated more heavy metals than herbivorous and omnivorous species.

Also, there are differences between the accumulation of heavy metals in marine and freshwater fishes. It was observed that the organs of freshwater fishes accumulate heavy metals more than marine fishes. This can be explained by the fact that freshwater fishes tend to lose salts and gain water, while marine fishes tend to do the opposite, so they are less exposed and vulnerable to heavy metal pollution (Nikinmaa 2014).

Potentially toxic elements were investigated by different authors in the tissues (feathers, kidney, liver, muscle) of waterfowl and terrestrial bird species of both sexes (Carpene et al. 1995; Cid et al. 2009; Grúz et al. 2019; Lehel et al. 2013; Malinga et al. 2010; Mazloomi et al. 2008; Mirsanjari et al. 2014; Sinkakarimi et al. 2018). However, no general trend could be drawn for sex differences based on the scientific literature.

Previously, toxic metals (As, Cd, Hg, Pb) in the feathers of 123 predatory birds (Long-eared owl [*Asio otus*], Barn owl [*Tyto alba*], Tawny owl [*Strix aluco*], Little owl [*Athene noctua*], Buzzard [Buteo buteo], Common kestrel [*Falco tinnunculus*], Eurasian sparrowhawk [*Accipiter nisus*]) were monitored by Grúz et al. (2019) in Hungary, but significant differences were not detected between genders. However, Mirsanjari et al. (2014) found in Iran that the concentrations of Cd, Cu, Pb and Zn in the feathers of male great cormorants (*Phalacrocorax carbo*) were significantly higher than female ones.

Statistical differences were not found in the concentrations of Hg and Pb in the liver of male and female cormorants (*Phalacrocorax carbo sinensis*) in Hungary (Lehel et al. 2013).

Pb and Cd concentrations in bone, pectoral muscle, liver, gonad and brain of piscivorous species such as Great grebe (*Podiceps major*), Neotropic cormorant (*Phalacrocorax brasilianus*) and, of omnivorous bird, Great kiskadee (*Pitangus sulphuratus*) were measured in Argentina, without differences in their levels between males and females (Cid et al. 2009).

Heavy metals such as Cd, Cu, Fe, Mn and Zn were monitored in the pectoral muscle, kidney, liver, brain, gonads, heart and feathers of Glaucous gulls (*Larus hyperboreus*) at Arctic region (Bjørnøya, Jan Mayen). The concentrations of Cu in the muscle and kidney were differed significantly only between the sexes (higher levels were detected in females) (Malinga et al. 2010).

Concentrations of Cd, Cu, Fe and Zn were investigated in the brain, gizzard, leg muscle, heart, breast muscle, intestine, liver and kidney of Moorhen (*Gallinula chloropus*), Black-headed gull (*Larus ridibundus*) and Black coot (*Fulica atra*) in Italy. Zn concentrations were higher in the leg muscle than in the pectoral muscle, but higher levels of Cu and Fe were detected in the breast muscle compared with thigh muscle. However, differences were not stated between genders (Carpene et al. 1995).

However, Hg concentrations between males and females were significantly different in the muscle and kidney of Caspian Sea common cormorant (*Phalacrocorax carbo*) in Iran (Mazloomi et al. 2008).

Sinkakarimi et al. (2018) studied the concentrations of Cd, chromium (Cr), iron (Fe), Pb and Zn in the kidney, liver and pectoral muscle of waterbirds, wintering Gadwall (*Anas strepera*) and Common Teal (*Anas crecca*) in Iran. Sex difference was noted only in the liver of males.

## Conclusion

The heavy and essential metal burden in the birds’ body is mostly determined by metal accessibility, food quality and environmental pollution presented in their nesting and feeding site.

Based on our results, the detected concentrations of these metals accumulated in pectoral and thigh muscle of great cormorants do not indicate that the habitat of the birds is contaminated by them on a level, which would cause toxicosis in the animals, or in humans, since there is a possibility that they consume the same fish species as the birds.

So, a routine monitoring of wildlife tissues in various areas is important to collect information about the metal burden of a region (rural or urban, land or water area).

## Data Availability

The datasets used and/or analysed during the current study are available from the corresponding author on reasonable request.

## References

[CR1] Aazami J, Esmaili-Sari A, Bahramifar N, Ghasempouri M, Savabieasfahani M (2011). Mercury in liver, kidney, feather and muscle of seabirds from major wetlands of the Caspian Sea, Iran. Bull Environ Contam Toxicol.

[CR2] Aazami J, KianiMeh N (2017). Survey of heavy metals in internal tissues of Great cormorant collected from southern wetlands of Caspian Sea, Iran. Environ Monit Assess.

[CR3] Alipour H, Pourkhabbaz A, Hassanpour M (2013). Assessing of heavy metal concentrations in the tissues of *Rutilus rutilus caspicus* and *Neogobius gorlap* from Miankaleh international wetland. Bull Environ Contam Toxicol.

[CR4] Anandkumar A, Nagarajan R, Prabakaran K, Rajaram R (2017). Trace metals dynamics and risk assessment in the commercially important marine shrimp species collected from the Miri coast, Sarawak, East Malaysia. Reg Stud Mar Sci.

[CR5] ATSDR (Agency for Toxic Substances and Disease Registry) (2000) Manganese. Atlanta, GA: U.S. Department of Health and Human Services, Public Health Service

[CR6] ATSDR (Agency for Toxic Substances and Disease Registry) (2012) Toxicological profile for Manganese. Atlanta, GA: U.S. Department of Health and Human Services, Public Health Service. 383-424

[CR7] Baker DH, Ammerman CB (1995) Zinc bioavailability. Pp.367-398 in Bioavailability of Nutrients for Animals. In: Ammerman CB, Baker DH, Lewis AJ (eds) Amino Acids, Minerals, and Vitamins. Academic Press, San Diego

[CR8] Bakonyi G, Juhász L, Kiss I, Palotás G (2003) Állattan (in Hungarian). Mezőgazda Kiadó, Hungary

[CR9] Bearhop S, Waldron S, Thompson D, Furness R (2000). Bioamplification of mercury in great skua Catharacta skua chicks: the influence of trophic status as determined by stable isotope signatures of blood and feathers. Mar Pollut Bull.

[CR10] Bloom NS (1992). On the chemical form of mercury in edible fish and marine invertebrate tissue. Can J Fish Aquat Sci.

[CR11] Blus LJ, Henny CJ, Hoffman DJ, Grove RA (1993). Accumulation and effects of lead and cadmium on wood ducks near a mining and smelting complex in Idaho. Ecotox.

[CR12] Burger J, Gochfeld M (2000). Metal levels in feathers of 12 species of seabirds from Midway Atoll in the northern Pacific Ocean. Sci Total Environ.

[CR13] Burger J, Gochfeld M (2000). Effects of lead on birds (Laridae): a review of laboratory and field studies. J Toxicol Environ Health Part B: Critical Reviews.

[CR14] Carpene E, Serra R, Isani G (1995). Heavy metals in some species of waterfowl of northern Italy. J Wildl Dis.

[CR15] Cheng Z, Lam CL, Mo WY, Nie XP, Choi WM, Man YB, Wong MH (2016). Food wastes as fish feeds for polyculture of low-trophic-level fish: bioaccumulation and health risk assessments of heavy metals in the cultured fish. Environ Sci Pollut Res.

[CR16] Cheung KC, Leung HM, Wong MH (2008). Metal concentrations of common freshwater and marine fish from the Pearl River Delta, South China. Arch Environ Contam Toxicol.

[CR17] Chiou PWS, Chen CL, Chen KL, Wu CP (1999). Effect of high dietary copper on the morphology of gastro-intestinal tract in broiler chickens. Asian-Australas J Anim Sci.

[CR18] Cid FD, Gatica-Sosa C, Antón RI, Caviedes-Vidal E (2009). Contamination of heavy metals in birds from Embalse La Florida (San Luis, Argentina). J Environ Monit.

[CR19] Cross FA, Hardy LH, Jones NJ, Barber RT (1973). Relation between total body weight and concentrations of manganese, iron, copper, zinc and mercury in white muscle of bluefish (*Pomatomus saltatrix*) and a bathyl-demersal fish (*Antimora rostrata*). J Fish Res Board Can.

[CR20] De Francisco N, Ruiz Troya JD, Aguera EI (2003). Lead and lead toxicity in domestic and free living birds. Avian Pathol.

[CR21] Decree of the Minister of Environment No. 13/2001 (2001): Decree of the Minister of Environment No. 13/2001 (KöM) on the Protected and Strictly Protected Plant and Animal Species, Strictly Protected Caves as well as on the Plant and Animal Species of Community Importance. Ministry of Environment, Budapest, Hungary

[CR22] EFSA (European Food Safety Authority) (2009). Scientific opinion of the panel on contaminants in the food chain on a request from the European Commission on cadmium in food. EFSA J.

[CR23] Eisler R (1988) Lead hazards to fish, wildlife and invertebrates: a synoptic review U.S. Fish and wildlife service, Washington

[CR24] Fehér G (2004) A háziállatok funkcionális anatómiája (in Hungarian). Mezőgazda Kiadó, Budapest

[CR25] Furness RW, Greenwood JJD (eds) (1993) Birds as monitors of environmental change. Champman & Hall. T. J. Press. Padstow, Cornwall

[CR26] Galey FD, Slenning BD, Anderson ML, Breneman PC, Littlefield ES, Melton LA, Tracy ML (1990). Lead concentrations in blood and milk from periparturient dairy heifers seven months after an episode of acute lead toxicosis. J Vet Diagn Investig.

[CR27] Gill F, Rand A, Storer RW et al. (2020) Bird. Encyclopædia Britannica. URL: https://www.britannica.com/animal/bird-animal. Accessed 17 Jan 2021

[CR28] Grúz A, Déri J, Szemerédy G, Szabó K, Kormos É, Bartha A, Lehel J, Budai P (2017). Monitoring of heavy metal burden in wild birds at eastern/north-eastern part of Hungary. Environ Sci Pollut Res.

[CR29] Grúz A, Mackle O, Bartha A, Szabó R, Déri J, Budai P, Lehel J (2019). Biomonitoring of toxic metals in feathers of predatory birds from Eastern Regions of Hungary. Environ Sci Pollut Res.

[CR30] Guitart R, Sachana M, Caloni F, Croubels S, Vandenbroucke V, Berny P (2010). Animal poisoning in Europe. Part 3: wildlife. Vet J.

[CR31] Harms RH, Buresh RE (1987). Influence of three levels of copper on the performance of turkey poults with diets containing two sources of methionine. Poult Sci.

[CR32] Honda K, Min BY, Ratsukawa R (1985). Heavy metal distribution in organs and tissues of the eastern great white egret *Egretta alba modesta*. Bull Environ Contam Toxicol.

[CR33] Horai S, Watanabe I, Takada H, Iwamizu Y, Hayashi T, Tanabe S, Kuno K (2007). Trace element accumulations in 13 avian species collected from the Kanto area, Japan. Sci Total Environ.

[CR34] Hosseini M, Nabavi SMB, Nabavi SN, Pour NA (2015). Heavy metals (Cd, Co, Cu, Ni, Pb, Fe, and Hg) content in four fish commonly consumed in Iran: risk assessment for the consumers. Environ Monit Assess.

[CR35] Houserova P, Hedbavny J, Matejicek D, Kracmar S, Sitko J, Kuban V (2005). Determination of total mercury in muscle, intestines, liver and kidney tissues of cormorant (*Phalacrocorax carbo*), great crested grebe (P*odiceps cristatus*) and Eurasian buzzard (*Buteo buteo*). Vet Med Czech.

[CR36] Hutton M (1981). Accumulation of heavy metals and selenium in three sea birds species from the United Kingdom. Environ Pollut Ser A.

[CR37] Jackson N, Stevenson MH (1981). A study of the effects of dietary added cupric oxide on the laying, domestic fowl and a comparison with the effects of hydrated copper sulfate. Br J Nutr.

[CR38] Jacob J, Pescatore AJ (2013) Avian muscular system. University of Kentucky College of Agriculture, Lexington, KY. Cooperative Extension Service. URL: https://poultry.extension.org/articles/poultry-anatomy/avian-muscular-system/ Accessed 17 Jan 2021

[CR39] Jia Y, Wang L, Qu Z, Wang C, Yang Z (2017). Effects on heavy metal accumulation in freshwater fishes: species, tissues and sizes. Environ Sci Pollut Res.

[CR40] Kim J, Shin J-R, Ko T-H (2009). Heavy metal distribution in some wild birds from Korea. Arch Environ Contam Toxicol.

[CR41] Kim J, Oh JM (2013). Assessment of trace metals in four bird species from Korea. Environ Monit Assess.

[CR42] Lavoie RA, Jardine TD, Chumchal MM, Kidd KA, Campbell LM (2013). Biomagnification of mercury in aquatic food webs: a worldwide meta-analysis. Environ Sci Technol.

[CR43] Lawrence AL, Mason RP (2001). Factors controlling the bioaccumulation of mercury and methylmercury by the estuarine amphipod Leptocheirus plumulosus. Environ Pollut.

[CR44] Lehel J, Laczay P (2011) Toxikológia (Az ökotoxikológus MSc szak hallgatói számára) (in Hungarian). Szent István Egyetemi Kiadó, Budapest

[CR45] Lehel J, Gál J, Faragó S, Berta E, Andrásofszky E, Fekete SG, Mándoki M, Budai P, Kormos É, Marosán M (2013). Evaluation of mercury and lead content in the liver of the cormorant (Phalacrocorax carbo sinensis) population of Kis-Balaton, Hungary. Acta Vet Hung.

[CR46] Lonnerdal B (2000). Dietary factors influencing zinc absorption. J Nutr.

[CR47] Malecki EA, Radzanowski GM, Radzanowski TJ, Gallaher DD, Greger JL (1996). Biliary manganese excretion in conscious rats is affected by acute and chronic manganese intake but not by dietary fat. J Nutr.

[CR48] Malik RN, Zeb N (2009). Assessment of environmental contamination using feathers of *Bubulcus ibis L*., as a biomonitor of heavy metal pollution. Pakistan Ecotoxicology.

[CR49] Malinga M, Szefer P, Gabrielsen GW (2010). Age, sex and spatial dependent variations in heavy metals levels in the Glaucous Gulls (Larus hyperboreus) from the Bjørnøya and Jan Mayen, Arctic. Environ Monit Assess.

[CR50] Markowski M, Kaliński A, Skwarska J, Wawrzyniak J, Bańbura M, Markowski J, Zieliński P, Bańbura J (2013). Avian feathers as bioindicators of the exposure to heavy metal contamination of food. Bull Environ Contam Toxicol.

[CR51] Mazloomi S, Esmaeili A, Ghasempoori SM, Omidi A (2008). Mercury distribution in liver, kidney, muscle and feathers of Caspian Sea Common Cormorant (Phalacrocorax carbo). Res J Environ Sci.

[CR52] Mirsanjari MM, Sheybanifar F, Arjmand F (2014). The study of Forest Hara Biosphere Reserve in coast of Persian Gulf and the importance of heavy metal accumulation; case study: feathers of great cormorant. Nusantara Biosci.

[CR53] Nam DH, Anan Y, Ikemoto T, Okabe Y, Kim EY, Subramanian A, Saeki K, Tanabe S (2005). Specific accumulation of 20 trace elements in great cormorants (*Phalacrocorax carbo*) from Japan. Environ Pollut.

[CR54] NRC (National Research Council) (1995) Manganese. In Nutrient Requirements of Laboratory Animals, 4th edn. National Academy Press, Washington, D.C.

[CR55] NRC (National Research Council) (2005) Mineral tolerance of animals 2nd edition. National Academic Press, Washington D.C

[CR56] Nguyen HL, Leermakers M, Kurunczi S, Bozo L, Baeyens W (2005). Mercury distribution and speciation in lake Balaton, Hungary. Sci Total Environ.

[CR57] Nikinmaa M (2014) An introduction to aquatic toxicology, 1st edn. Academic Press, Cambridge, MA, USA, p 24 ISBN 978-0-12-411574-3

[CR58] O’Halloran TV (1993). Transition metals in control of gene expression. Science.

[CR59] Ortego J, Jiménez M, Díaz M, Rodríguez RC (2006). Mercury in feathers of nestling eagle owls, Bubo bubo l., and muscle of their main prey species in Toledo province, Central Spain. Bull Environ Contam Toxicol.

[CR60] Perugini M, Visciano P, Manera MI, Zaccaroni A, Olivieri V, Amorena M (2014). Heavy metals (As, Cd, Hg, Cu, Zn, Se) concentrations in muscle and bone of four commercial fish caught in the central Adriatic Sea, Italy. Environ Monit Assess.

[CR61] Pesti GM, Bakalli RI (1996). Studies on the feeding of cupric sulfate pentahydrate and cupric citrate to broiler chickens. Poult Sci.

[CR62] Saeki K, Okabe Y, Kim EY, Tanabe S, Fukuda M, Tatsukawa R (2000). Mercury and cadmium in common cormorants (Phalacrocorax carbo). Environ Pollut.

[CR63] Scheuhammer A, Meyer M, Sandheinrich M, Murray M (2007). Effects of environmental methylmercury on the health of wildbirds, mammals, and fish. Ambio.

[CR64] Sinkakarimi M-H, Binkowski LJ, Hassanpour M, Rajaei G, Ahmadpour M, Levengood JM (2018). Metal concentrations in tissues of Gadwall and Common Teal from Miankaleh and Gomishan International Wetlands, Iran. Biol Trace Elem Res.

[CR65] Underwood EJ, Suttle NF (1999) The mineral nutrition of livestock, 3rd edn. CABI Publishing, New York

[CR66] Varnai VM, Piasek M, Blanusa M, Saric MM, Simic D, Kostial K (2001). Calcium supplementation efficiently reduces lead absorption in suckling rats. Pharmacol Toxicol.

[CR67] Waldner C, Checkley S, Blakley B, Pollock C, Mitchell B (2002). Managing lead exposure and toxicity in cow-calf herds to minimize the potential for food residues. J Vet Diagn Investig.

[CR68] Wilgus HS, Patton AR (1939). Factors affecting manganese utilization in the chicken. J Nutr.

[CR69] Yasmeen R, Muhammad HA, Bokhari SS, Rafi U, Shakoor A, Qurashi AW (2019). Assessment of heavy metals in different organs of cattle egrets (*Bubulcus ibis*) from a rural and urban environment in Pakistan. Environ Sci Pollut Res.

